# Targeted deep sequencing of plasma circulating cell-free DNA reveals *Vimentin* and *Fibulin 1* as potential epigenetic biomarkers for hepatocellular carcinoma

**DOI:** 10.1371/journal.pone.0174265

**Published:** 2017-03-23

**Authors:** Reetta Holmila, Athena Sklias, David C. Muller, Davide Degli Esposti, Paule Guilloreau, James Mckay, Suleeporn Sangrajrang, Petcharin Srivatanakul, Pierre Hainaut, Philippe Merle, Zdenko Herceg, Andre Nogueira da Costa

**Affiliations:** 1 Epigenetics group, International Agency for Research on Cancer (IARC), Lyon, France; 2 Genetic Epidemiology group, International Agency for Research on Cancer (IARC), Lyon, France; 3 Croix-Rousse Hospital, Lyon, France; 4 Genetic Cancer Susceptibility group, International Agency for Research on Cancer (IARC), Lyon, France; 5 Molecular Epidemiology Section, National Cancer Institute, Bangkok, Thailand; 6 Institut Albert Bonniot, INSERM Unité 823, La Tronche, France; 7 UMR INSERM 1052, CRCL, Lyon, France; 8 Molecular mechanisms and biomarkers group, International Agency for Research on Cancer (IARC), Lyon, France; Johnson & Johnson Medical, CHINA

## Abstract

Hepatocellular carcinoma (HCC) is the second most common cause of cancer death worldwide, but is still lacking sensitive and specific biomarkers for early diagnosis and prognosis. In this study, we applied targeted massively parallel semiconductor sequencing to assess methylation on a panel of genes (*FBLN1*, *HINT2*, *LAMC1*, *LTBP1*, *LTBP2*, *PSMA2*, *PSMA7*, *PXDN*, *TGFB1*, *UBE2L3*, *VIM* and *YWHAZ*) in plasma circulating cell-free DNA (cfDNA) and to evaluate the potential of these genes as HCC biomarkers in two different series, one from France (42 HCC cases and 42 controls) and one from Thailand (42 HCC cases, 26 chronic liver disease cases and 42 controls). We also analyzed a set of HCC and adjacent tissues and liver cell lines to further compare with ‘The Cancer Genome Atlas’ (TCGA) data. The methylation in cfDNA was detected for *FBLN1*, *PSMA7*, *PXDN* and *VIM*, with differences in methylation patterns between cases and controls for *FBLN1* and *VIM*. The average methylation level across analyzed CpG-sites was associated with higher odds of HCC for *VIM* (1.48 [1.02, 2.16] for French cases and 2.18 [1.28, 3.72] for Thai cases), and lower odds of HCC for *FBLN1* (0.89 [0.76, 1.03] for French cases and 0.75 [0.63, 0.88] for Thai cases). In conclusion, our study provides evidence that changes in *VIM* and *FBLN1* methylation levels in cfDNA are associated with HCC and could represent useful plasma-based biomarkers. Also, the potential to investigate methylation patterns in cfDNA could bring new strategies for HCC detection and monitoring high-risk groups and response to treatment.

## Introduction

Liver is the fifth most common organ site for cancer in men and the ninth in women. The liver cancer is frequently diagnosed at a late stage and has a poor prognosis (overall ratio of mortality to incidence of 0.95) making it the second most common cause of death from cancer globally. Hepatocellular carcinoma (HCC), which originates from hepatocytes, represents over 80% of primary liver cancer cases and is the third most frequent cause of cancer-related death worldwide, with considerable geographic variation in rates and etiology [1,2,3]. Most areas of high incidence are in low-resource countries accounting for about 80% of the new liver cancer cases worldwide [1].

In the low-resource contexts, liver biopsies are often not feasible and the diagnosis of HCC commonly relies on a combination of clinical symptoms, ultrasound, and analysis of α-fetoprotein (AFP) levels in serum [4]. However, AFP remains unsatisfactory for diagnosis and screening as such high levels of AFP are detected only in a subset of patients and AFP levels above 100 ng/mL may be observed in some patients with non-cancer chronic liver diseases [5]. For these reasons, the American Association for the Study of Liver Diseases (AASLD) does not recommend the use of AFP testing as a part of the diagnostic criteria for HCC and considers imaging techniques as more reliable for diagnosis of HCC [6]. Nevertheless, due to the lack of alternative plasma marker easily applicable in low-resource context, AFP is still widely used for HCC diagnosis with a cutoff value of 200 ng/mL proposed by the Asian Pacific Association for the Study of the Liver [3]. Other markers have also been proposed for diagnosis of HCC, including lens culinaris-reactive AFP (AFP-L3), HCC-specific gamma- glutamyltransferase (HS-GGT) and glypican-3 (GPC3) [7]. To date, most of these markers have not shown better performance for detection of HCC than AFP or AFP combined with ultrasound [4].

HCC carcinogenesis is a multi-step process that usually arises in background chronic metabolic, inflammatory and/or infectious liver disease [2]. Epigenetic mechanisms have been reported to play an important role in the development of HCC and aberrant DNA methylation patterns have been found in the earliest stages of hepatocarcinogenesis and increasing during the tumor progression [8]. We recently showed that massively parallel semiconductor deep sequencing could be used to detect and analyze methylation changes in circulating cell-free DNA (cfDNA) [9]. In the present study, we applied this targeted analysis to investigate cfDNA methylation in plasma specimens from HCC case-control studies from France and Thailand, two regions with differing disease prevalence and etiology. The aim of this study was to assess cfDNA methylation in a panel of genes and evaluate their potential as novel biomarkers for HCC diagnosis. We also analyzed a set of HCC and adjacent tissue samples as well as different liver cell lines to further compare with ‘The Cancer Genome Atlas’ (TCGA) data in order to explore the origin of methylation patterns in cfDNA methylation.

## Materials and methods

### Patient characteristics

Plasma specimens were collected and processed as previously described [10]. In France, blood specimens and tissue samples were obtained from hospital-based controls and from patients with HCC recruited at Hôpital Croix-Rousse in Lyon (France) between 2011 and 2012 and HCC was diagnosed according to AASLD guidelines and Barcelona Clinic Liver Cancer staging system [11]. In Thailand, specimens were obtained from patients with HCC, with chronic liver disease (including patients with chronic active hepatitis B) and hospital-based controls recruited at the Cancer Control Unit of the National Cancer Institute of Thailand, Bangkok between 2008 and 2010. Differential diagnosis of HCC versus cholangiocarcinoma was established by a combination of clinical examination, imaging using ultrasonography, computerized tomography (CT) or Magnetic Resonance Imaging (MRI), biochemistry (AFP and liver function enzymes testing) and histological confirmation on a small subset of patients from whom needle biopsies were available. A total of 194 plasma samples were utilized for methylation analysis of which 42 HCC patients and 42 hospital-based controls without liver symptoms from France (S1 Table) and 42 HCC patients, 26 chronic liver disease patients and 42 hospital-based controls from Thailand (S2 Table). From France, also nine tumor and paired adjacent non-tumor tissue samples were acquired from a different set of HCC patients from the same case series (S3 Table). All adjacent non-tumor tissues were cirrhotic. In addition to tumor samples, six liver cell lines were analysed: HepG2, HepG2.2.15, Hep3B, PLC/PRF/5, Mahlavu and HepaRG. The conditions for the cell culture are described in the supplementary material (S1 File). Written consent was obtained from all participants and all steps of the study (patient recruitment, consent procedure, sample collection and processing, methylation analysis and data analysis) were approved by the Institutional Review Boards of the Thailand National Cancer Institute and the International Agency for Research on Cancer.

### DNA extraction and bisulfite treatment

The cfDNA was extracted from 1mL of plasma using the QIAamp circulating nucleic acid kit (Qiagen, Valencia, CA, USA) with the QIAvac 24 Plus vacuum manifold, following manufacturer’s instructions. CfDNA was quantified by the Quant-iT PicoGreen dsDNA assay (Life Technologies), the mean DNA concentration was for controls from France 0.16 ng/μl (0.04–1.33 ng/μl) and from Thailand 0.35 ng/μl (0.01–0.69 ng/μl), for the chronic liver disease patients 0.32 ng/μl (0.10–0.66 ng/μl) and for the HCC patients from France 0.37 ng/μl (0.1–2.76 ng/μl) and from Thailand 1.08 ng/μl (0.10–3.14 ng/μl). DNA from tissue samples was extracted using QIAamp DNA Mini Kit (Qiagen) and from cell pellets using Qiagen AllPrep DNA/RNA Mini Kit (Qiagen) following manufacturer’s instructions and quantified by NanoDrop (Thermo Fisher Scientific). From all samples, 5–10 ng of DNA was used for the bisulfite transformation by EZ DNA Methylation-Gold Kit (Zymo Research) and the manufacturer’s protocol was followed.

### Primer design and amplification of targets

The primers were designed for one strand using Methprimer software [12] with default parameters to amplify sequences of 70 to 150 bp spanning the proximal promoter CpG island regions of *Fibulin 1* (*FBLN1)*, *Histidine Triad Nucleotide-Binding Protein 2 (HINT2)*, *Laminin*, *gamma 1 (LAMC1)*, *Latent-transforming growth factor beta-binding protein 1 (LTBP1)*, *Latent transforming growth factor beta binding protein 2 (LTBP2)*, *Proteasome subunit alpha type-2* (*PSMA2)*, *Proteasome subunit alpha type-7 (PSMA7*), *Peroxidasin Homolog* (*PXDN)*, *Transforming Growth Factor Beta-1 (TGFB1)*, *Ubiquitin-conjugating enzyme E2 L3* (*UBE2L3)*, *Vimentin (VIM*) and *Tyrosine 3-monooxygenase/tryptophan 5-monooxygenase activation protein*, *zeta (YWHAZ)*, (see S4 Table for primer sequences and targeted regions). The methylation profiles of targets were not known at the time of the primer design. The targets were chosen to evaluate potential HCC biomarkers ([10], on-going work and the literature [13,14,15,16,17,18,19,20]). Multiplex PCR reactions were designed using the Multiplx online tool (http://bioinfo.ebc.ee/multiplx) and the resulting three primer mixes were tested for incompatibilities with PriDimerChek (http://biocompute.bmi.ac.cn/MPprimer/primer_dimer.html). For target amplification, 1–2 ng of bisulfite treated cfDNA were used in PCR reactions with the GoTaq® HotStart DNA polymerase (Promega Corporation) and with the program: 30s at 94°C, 3 cycles of 30s at 58.5°C, 30s at 72°C, subsequently, the annealing temperature was decreased 0.5°C every 3 cycles until reaching 55.5°C; then 15 cycles of 30s at 94°C, 45s at 50°C, 1 min at 72°C, and a final extension of 10 min at 72°C.

### Sequencing by Ion Torrent™ PGM sequencer and methylation analysis

To verify the success of the PCR amplification and adjust for the quantity of reactions, 1 μL of PCR reaction was loaded on a gel and the adjusted quantities were pooled by sample for equalizing target representation. Pools were purified with Agencourt AMPure beads with a ratio of PCR products of 2:1 (Beckman Coulter Incorporated) and quantified by Qbit (Invitrogen Corporation). Library preparation was done using 30 ng of pooled DNA and the NEBNext Fast DNA Library Prep Set for Ion Torrent (New England Biolabs) following manufacturer’s instructions. Individual barcodes (designed in-house and produced by Eurofins MWG Operon) were ligated to each pool and followed by six PCR cycles and gel purification for sequencing. The libraries were sequenced with the Ion Torrent™ PGM sequencer (Life Technologies) at deep coverage (minimum 100 reads, mean read depth 2500, standard deviation 2700) using the Ion OneTouch 200 Template Kit (versions v1 and v2) DL and Ion PGM Sequencing 200 Kit v2 with the 316 chip kit (Life Technologies), following the manufacturer’s instructions. The cases and controls were randomly distributed across different batches and analyzed blinded to the case-control status.

The sequencing reads were aligned to the bisulfite-converted target genomic regions of all genes (reference hg19) with the Ion Torrent Suite V3.4.2. The aligned BAM files were visualized by Integrative Genomics Viewer (IGV) 2.2 (Broad Institute) [21]. A hotspot BED file containing the position of the queried CpG sites and control cytosine bases (to verify the bisulfite conversion efficiency) in the target regions was used to extract the read counts for CpG sites and control cytosines using the HID SNP Genotyper Plugin (v3.0.0) on the Ion Torrent Suite. The methylation index was counted as the percentage of cytosine reads of the total of cytosine and thymine reads of each CpG and control cytosine site. Samples with methylation index values in the non-CpG control cytosines above 1% were considered as not fully bisulfite converted and were excluded from the analysis [9].

### Statistical analysis

For each participant, a methylation score was calculated for each gene by averaging the observed methylation percentage for the CpG sites within each gene. Mean methylation proportions and 95% confidence intervals were calculated separately for HCC patients, chronic liver disease patients and controls, as well as chronic liver disease patients and controls combined (non-HCC cases). Logistic regression was used to estimate odds ratios and 95% confidence intervals for a 1 percentage point increment in methylation score. Analyses were conducted separately for the Thailand and France case-control studies. All analyses were conducted using R version 3.1.2 [22].

### Analysis of the The Cancer Genome Atlas (TCGA) methylation data

Illumina HumanMethylation 450k level 1 data on 47 HCC patients were downloaded from ‘The Cancer Genome Atlas’ (TCGA, http://tcga-data.nci.nih.gov/). In total, 94 tumor and adjacent non-tumor samples were selected and processed into a paired analysis. Raw data (idat) were filtered out for low quality data, normalized using the BMIQ method [23] and annotated with hg18 ending with a set of 41 usable pairs. β-values for *FBLN1* and *VIM* were extracted from this subset. All analyses were performed using R (version 3.1.0) using wateRmelon and minfi packages [24,25].

## Results

Methylation in circulating cfDNA was detected by massively parallel bisulfite sequencing for *FBLN1*, *PSMA7*, *PXDN* and *VIM*, whereas for *HINT2*, *LAMC1*, *LTBP1*, *LTBP2*, *PSMA2*, *TGFB1*, *UBE2L3* and *YWHAZ* methylation was below the detection limit (1% of methylation [9]). When comparing HCC cases with controls, we observed differences in methylation levels of *FBLN1* and *VIM*, whereas no differences were observed for *PSMA7* and *PXDN* (S1 Fig) findings for *FBLN1* and *VIM* are further discussed below.

### Methylation of FBLN1 and VIM in plasma DNA

**Fig 1:**
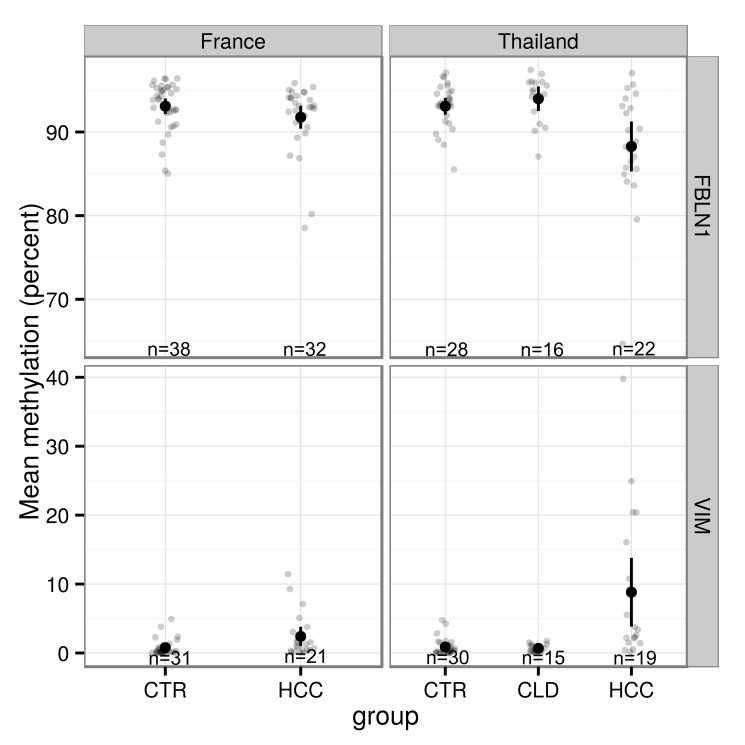
Mean methylation proportions and 95% confidence intervals for *FBLN1* and *VIM* in France and in Thailand. HCC = hepatocellular carcinoma, CTR = control, CLD = chronic liver disease.

**Fig 2:**
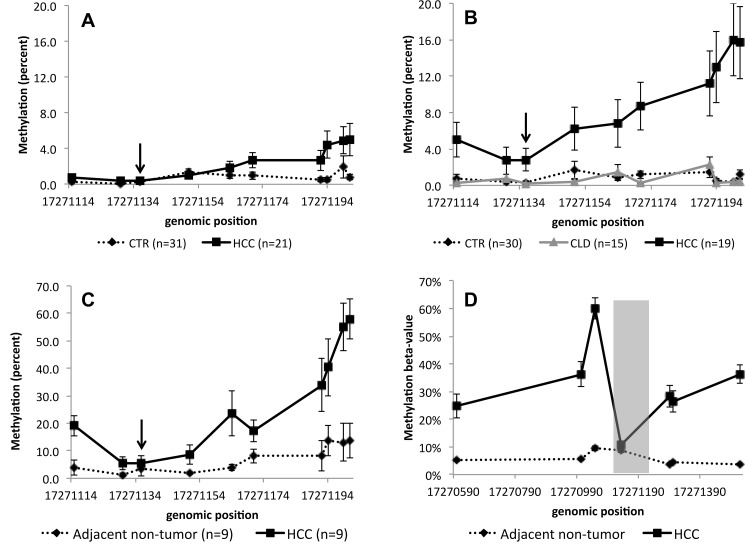
*VIM* methylation in cfDNA. Cases from France (A) and Thailand (B), in tissue (C) (the arrows show the CpG-site analysed in TCGA data) and in TCGA data (D) (the grey area represents the area analyzed by massively parallel sequencing in this study). CTR: controls, HCC: hepatocellular carcinoma patients and CLD: chronic liver diseases. The error bars represent standard error of mean.

**Fig 3:**
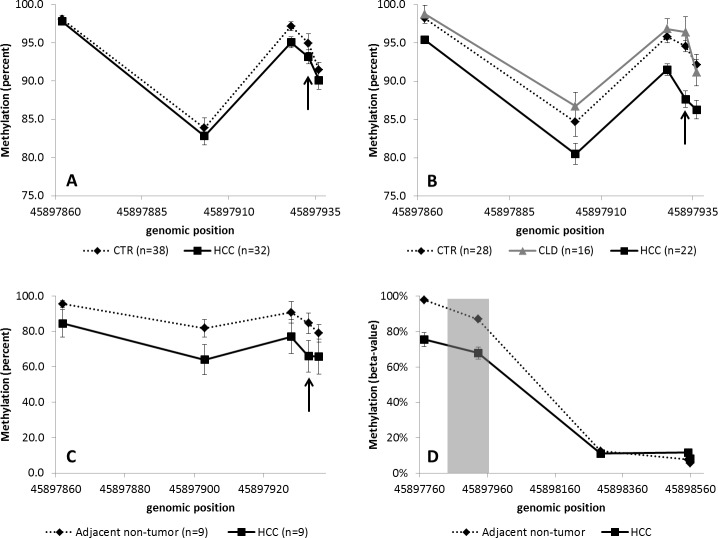
*FBLN1* methylation in cfDNA. Cases from France (A) and Thailand (B), in tissue (C) (the arrows show the CpG-site analysed in TCGA data) and in TCGA data (D) (the grey area represents the area analyzed by massively parallel sequencing in this study). CTR: controls, HCC: hepatocellular carcinoma patients and CLD: chronic liver diseases. The error bars represent standard error of mean.

**Fig 4:**
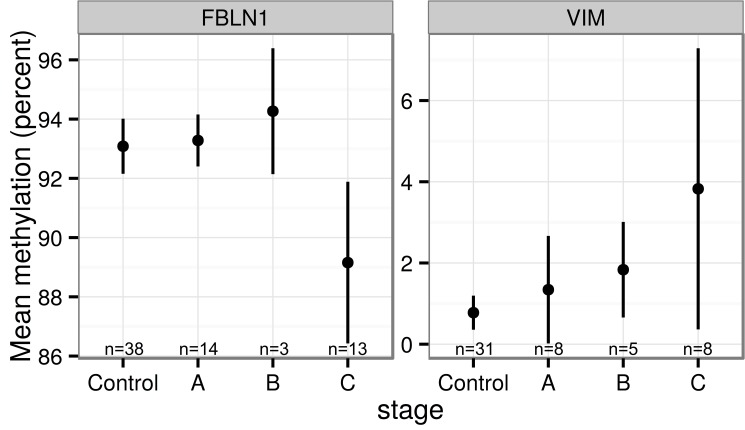
Mean methylation proportions and 95% confidence intervals for HCC cases by tumor stages. A, B and C (Barcelona Clinic Liver Cancer staging system) and controls from France.

After exclusion of samples for technical reasons, such as incomplete bisulfite conversion or failed amplification, the methylation levels were analyzed for *FBLN1* for 38 controls and 32 HCC patients from France and for 28 controls, 16 chronic liver disease patients and 22 HCC patients from Thailand, and for *VIM* for 31 controls and 21 HCC patients from France and for 30 controls, 15 chronic liver disease patients and 19 HCC patients from Thailand. As stated above, *FBLN1* and *VIM* methylation patterns were found to be different between controls and HCC patients for both France and Thailand (Figs 1–3). It is of note that for chronic liver disease patients from Thailand, the methylation patterns detected were similar to those observed in the controls (Figs 2 and 3). There appeared to be no clear association between the methylation levels of these genes and the levels of AFP in plasma (S2 Fig). Higher mean methylation levels were associated with greater odds of HCC for *VIM*, and lower odds of HCC for *FBLN1*, though the latter association was only of borderline significance in the France series (Table 1). Adjusting for age and sex did not materially affect these estimates. As information on HCC clinical stage (according to the Barcelona Clinic Liver Cancer staging system) and etiology were available only for the French cases we examined mean methylation levels by stage and etiology in this series. As shown in Fig 4, although the results remained statistically borderline or insignificant due to the small numbers and variation between cases, cases with higher HCC stage appeared to have a consistently higher methylation levels for *VIM* and lower methylation levels for FBLN1. Further analysis of DNA methylation data stratified by associated etiological factors showed no significant differences between the strata (S3 Fig).

**Table 1:** Odds ratios (OR) of hepatocellular carcinoma and 95% confidence intervals (CI) for a percentage point increment in methylation.

Gene	Country	OR [95% CI]	p
*FBLN1*	France	0.89 [0.76, 1.03]	1.03E-01
*FBLN1*	Thailand	0.75 [0.63, 0.88]	2.82E-05
*VIM*	France	1.48 [1.02, 2.16]	9.43E-03
*VIM*	Thailand	2.18 [1.28, 3.72]	7.74E-08

### Methylation of FBLN1 and VIM in tissue samples and TCGA data

*FBLN1* and *VIM* methylation was also studied in liver tissues (including HCC tissue and paired adjacent non-tumor tissue) from separate nine cases from the French case series. The mean of the differences between paired tumor and non-tumor methylation percentages was -15% (95% CI [–32, 2]) for *FBLN1* and 19% (95% CI [7, 32]) for *VIM*, consistent with the changes in methylation detected in cfDNA. The pattern of methylation in cfDNA from controls was similar to the methylation in adjacent non-tumor tissue samples (Figs 2 and 3). Comparison with the methylation data available in the TCGA for *FBLN1* and *VIM* methylation in HCC confirmed the differential methylation patterns of these genes in HCC and in control tissues (Fig 2D, Fig 3D).

### Methylation of FBLN1 and VIM in liver cell lines with different TP53 mutation status

**Fig 5:**
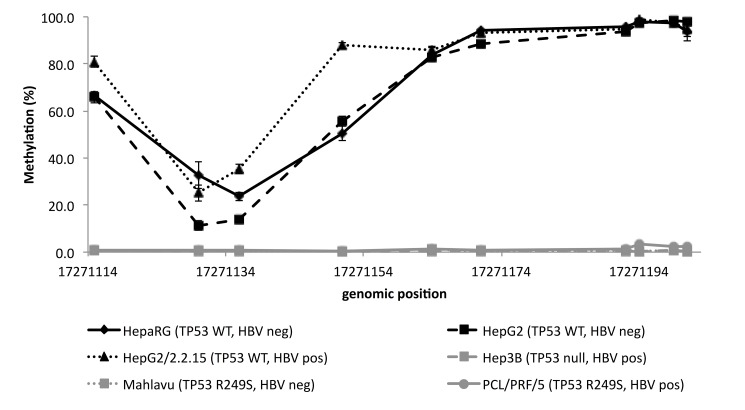
*VIM* methylation in liver cell lines with different *TP53* mutation and HBV–status. WT = wild type, R249S = missense mutation in the codon 249 of TP53.

As HBV alters the methylation and interacts with *TP53* in HCC, we next analyzed the methylation of *FBLN1* and *VIM* in six liver cell lines (HepaRG, HepG2, HepG2/2.2.15, Hep3B, Mahlavu and PCL/PRF/5) with different *TP53* mutation and HBV-status. For *FBLN1* the overall methylation pattern resembled the one detected in plasma and tissue samples, with the exception of HepG2 and HepG2/2.2.15 that had lower mean methylation levels (10.1% and 56.2% respectively), whereas for *VIM* the methylation pattern seemed to vary with *TP53* mutation status. For the cell lines with wild type *TP53* (HepaRG, HepG2 and HepG2/2.2.15), the methylation followed the pattern detected in plasma and tumor tissue samples. However, for cell lines that were deficient or mutated for *TP53* (Hep3B, Mahlavu and PCL/PRF/5), *VIM* methylation was below the detection limit or very low (Fig 5).

### Methylation of VIM and TP53 mutations

As the *VIM* methylation in liver cell lines appeared to be associated with *TP53* mutation status, we analyzed the HCC tissues for *TP53* mutations in exons 4–10. *TP53* mutations were detected in two out of 9 tumors (22%). HCC case 9T carried p.E271V missense mutation that according to the *TP53* database [26] is causing p53 to be non-functional for DNA-binding, and HCC case 10T carried p.L137Q missense mutation that according to the *TP53* database does not change the functionality of the p53 protein. The mean methylation level for 9T was 6.1%, whereas for 10T it was 52.3%. Also in the TCGA dataset, *VIM* methylation on the CpG-sites appeared to depend on the *TP53* mutation status and type. Especially in early HCC tumor stages, stage I and II, the tumors with *TP53* missense mutations appeared to have lower levels of *VIM* methylation than the tumors with wild type *TP53* (S4 Fig), but the association remained statistically non-significant, possibly due to relatively small numbers.

## Discussion

Epigenetic alterations, including global DNA hypomethylation and specific CpG island hypermethylation linked with inactivation of tumor suppressor genes, have been detected in many types of human cancers, including HCC. These alterations can play a role in early carcinogenesis and therefore could potentially be useful as biomarkers for early detection, prognostic and prediction of therapy responses [2,27]. Recently, there has been a growing interest in the predictive and prognostic value of detecting tumor-specific genetic and epigenetic alterations in cfDNA from biofluids, such as plasma, serum or urine [28,29], as biofluids are more easily available compared with primary tissues and can be analyzed regardless of the patient’s condition and disease progression. Since HCC carcinogenesis has a strong epigenetic component, there is interest in studying epigenetic changes as potential biomarkers for HCC, especially since HCC still lacks sensitive and robust biomarkers [7]. Specifically, the possibility of detecting changes in HCC methylation in cfDNA ahead of conventional tumor diagnosis may help to develop new strategies for early detection [30].

The aim of this study was to evaluate whether methylation changes related to HCC could be detected in cfDNA using targeted massively parallel deep sequencing. A previous study demonstrated that this technique could reproducibly detect and measure methylated cfDNA fragments in the plasma and requires very little DNA for the analysis and therefore has the advantage compared with the methylation arrays [9]. Here, the cfDNA methylation was analyzed in controls and HCC cases in two different series, one from France and one from Thailand, corresponding to two different epidemiological contexts for the development of HCC. The methylation in cfDNA was detected for *FBLN1*, *PSMA7*, *PXDN* and *VIM* and the differences in the methylation patterns were found for *FBLN1* and *VIM* when comparing HCC cases to controls in both series, whereas for *PSMA7* and *PXDN* no differences were seen. *FBLN1* and *VIM* methylation levels did not appear to correlate with AFP levels or with distinct etiological factors. It is of note that the differences in methylation between HCC cases and controls were larger in the Thailand series compared to the France series. This may be attributable to the pathologically more advanced stage of HCC at diagnosis of the cases from the Thailand series, due to the later detection of HCC in a context of limited access to diagnostic facilities [31].

*VIM* and *FBLN1* are key components of the extracellular matrix (ECM) that are involved in epithelium to mesenchyme transition (EMT). *VIM* is a member of intermediate filament protein family involved in cytoskeleton structure regulation associated with physiological and pathological changes [32]. *VIM* has also been associated with signaling transduction and has been proposed to transfer information from the ECM to the nuclei, which is an important step in the EMT that leads to loss of cellular adhesion and invasion of tumor cells [33]. In human cancers, aberrant methylation of *VIM* has been shown in colorectal cancer [34], gastric cancer [35], bladder cancer [36], pancreatic cancer [37], cervical cancer [38] and breast cancer [39]. Detection of *VIM* methylation from DNA in serum, urine or feces has already been proposed as a biomarker for colorectal, gastric and bladder cancers [36,40,41,42,43]. In HCC, aberrant methylation of *VIM* has been suggested to be associated with primary HCC and correlated with clinicopathological variables, including alpha-fetoprotein levels and maximal tumor size [20]. *FBLN1* is a secreted glycoprotein that is found in association with ECM structures including fibronectin and elastin containing fibers and basement membranes and it has been implicated in cellular transformation and tumor invasion [44,45]. *FBLN1* has been reported to act as a tumor suppressor gene and to be regulated by promoter hypermethylation in gastric and prostate cancer [46,47], and in renal cell carcinoma and bladder cancer the *FBLN1* promoter hypermethylation has been shown to correlate with gene expression and tumor stage [44,48]. Also in HCC, the promoter hypermethylation of *FBLN1* has been described and shown to be associated with reduced expression of *FBLN1* mRNA, advanced stage HCC, multiple tumors and increased tumor size [17]. In our study the *FBLN1* methylation was described to be lower among the HCC cases than controls, this is probably due to the different area of proximal promoter analyzed compared to the other studies. The altered methylation levels of *VIM* and *FBLN1* may be a consequence of remodeling of tissue structures associated with altered signaling transduction involved in hepatocyte/matrix interactions in EMT that could influence ECM protein capacity to contribute or regulate migration, adhesion and invasion of cells during a liver fibrogenic process and subsequent development of HCC.

As cfDNA can in principle originate from different tissue and organ locations, we also sought to analyze *FBLN1* and *VIM* methylation in liver tissues, including HCC tissue and adjacent non-tumor tissues, of patients recruited in the French series. The patterns of methylation detected in tissues were similar to the one detected in cfDNA, which were both analyzed by us using massively parallel sequencing methodology, and were consistent with the TCGA data based on Illumina methylation arrays. Methylation levels were higher in HCC tissues compared with cfDNA from HCC patients as would be expected, since the cfDNA originating from HCC most likely represents only a fraction of the total cfDNA. The pattern of methylation in adjacent non-tumor tissue samples was similar to the methylation in cfDNA from controls. These data provide indirect evidence that the methylation differences detected in cfDNA are reflecting those in tumor cell DNA, even though we did not have the access to both plasma and tissue samples from the same HCC patients. Of note, according to TCGA dataset, important differences in methylation status between cases and controls are associated with a 100bp region of *VIM* located immediately upstream of the region we analyzed by massively parallel sequencing. Thus, including this region in analysis would be expected to reveal even larger differences in *VIM* methylation than those reported here.

In liver cell lines, the *FBLN1* methylation pattern was similar to the cfDNA and tissue, whereas for *VIM* different methylation patterns were found. In cell lines harboring wild type *TP53*, the *VIM* methylation pattern was similar to the one detected in cfDNA and in tissues, even though overall methylation levels were much higher (near 100%). Strikingly in the cell lines with *TP53* mutation, either missense mutation or null *TP53* genotype, the *VIM* methylation was very low (near 0%). This prompted us to analyze the *TP53* mutation status also in the HCC tissues. We characterized two tumors in our limited study set that harbored a missense mutation, one with predicted transcriptionally functional mutation most likely representing a “passenger” genetic event in the tumor, and the other with predicted non-functional mutation likely to represent “driver” mutational event. The HCC tumor with predicted functional *TP53* showed similar methylation pattern to the tumors with wild type *TP53*, whereas the tumor with predicted non-functional *TP53* had very low levels of methylation. These observations seem to suggest that low methylation of *VIM* is associated with impaired p53 function. We also compared the *TP53* mutations to the *VIM* methylation in the TCGA dataset and overall, the HCC tumors harboring *TP53* mutation seemed to have lower levels of *VIM* methylation than the tumors with wild type *TP53*, especially in the earlier tumor stages. Nevertheless, these results remain preliminary due to the limited numbers of cases with *TP53* mutations both in our sample set and TCGA data sets.

Altogether, our study provides evidence that measuring methylation levels of *VIM* and *FBLN1* in plasma cfDNA may be effective for biomarker-based detection and follow-up of HCC, contributing to novel strategies for improved diagnosis accuracy and patient surveillance. The main limitations of this study are the heterogeneous stages at which HCC was diagnosed and differing basis for patient recruitment and diagnosis between the two different case series. Also, we did not define sensitivity and specificity even though they are important measures that in general partly characterize the performance of diagnostic biomarkers. The calculation of sensitivity and specificity requires definition of a cut-point which defines high- and low-probability groups which in turn necessitates a full decision theoretic analysis, that in turn takes into account the baseline risk in the target population, as well as the relative benefits and harms of false positive and false negative results. Such an analysis for methylation of *VIM* and *FBLN1* is premature at this stage, and beyond the scope of this manuscript. We have shown that the average methylation level across analyzed CpG-sites in cfDNA was associated with higher odds of HCC for *VIM* (1.48 [1.02, 2.16] for French cases and 2.18 [1.28, 3.72] for Thai cases), and lower odds of HCC for *FBLN1* (0.89 [0.76, 1.03] for French cases and 0.75 [0.63, 0.88] for Thai cases) which represents a critical first step in the evaluation of these as potential biomarkers. Larger validation studies, including prospective studies on groups of participants with different risk factors and patterns of chronic liver disease, would be needed, allowing for detailed analyses of association with tumor occurrence, size and clinical parameters. It would also be important to evaluate how *VIM* and *FBLN1* methylation patterns vary in liver metastases. Nevertheless, in the context of diagnosis and potential early detection of HCC, detecting the changes to *VIM* and *FBLN1* methylation patterns in plasma cfDNA holds a promise for improved diagnosis and disease progression monitoring.

## Supporting information

S1 Fig Mean methylation proportions and 95% confidence intervals by study sample for *PSMA7* and *PXDN*. HCC = hepatocellular carcinoma, CTR = control, CLD = chronic liver disease.
(TIFF)

S2 Fig Methylation proportions by AFP value. (TIFF)

S3 Fig Methylation levels by different etiologies. HCV = Hepatitis C virus, OH = alcohol.
(TIFF)

S4 Fig *VIM* methylation in the HCC tumors in the TCGA data by the *TP53* mutation status. A) *VIM* methylation compared with overall *TP53* mutation status, B) *VIM* methylation by *TP53* mutation in stage I tumors, C) *VIM* methylation by *TP53* mutation in stage II tumors, D) *VIM* methylation by *TP53* mutation in stage III tumors. MUT = mutated, WT = wild type.
(TIFF)

S1 Table Characteristics of the French series. (DOCX)

S2 Table Population characteristics of the Thailand case-control study (DOCX)

S3 Table Characteristics of the HCC tissue samples. (DOCX)

S4 Table Primer and product sequences for the sequencing targets. (DOCX)

S1 File Cell lines and cell culture. (DOCX)
