# Supplementary figures and images for: Targeted deep sequencing of plasma circulating cell-free DNA reveals Vimentin and Fibulin 1 as potential epigenetic biomarkers for hepatocellular carcinoma
Source: PLoS One. 2017 Mar 23;12(3):e0174265. doi: 10.1371/journal.pone.0174265 (PMC5363871; doi:10.1371/journal.pone.0174265)

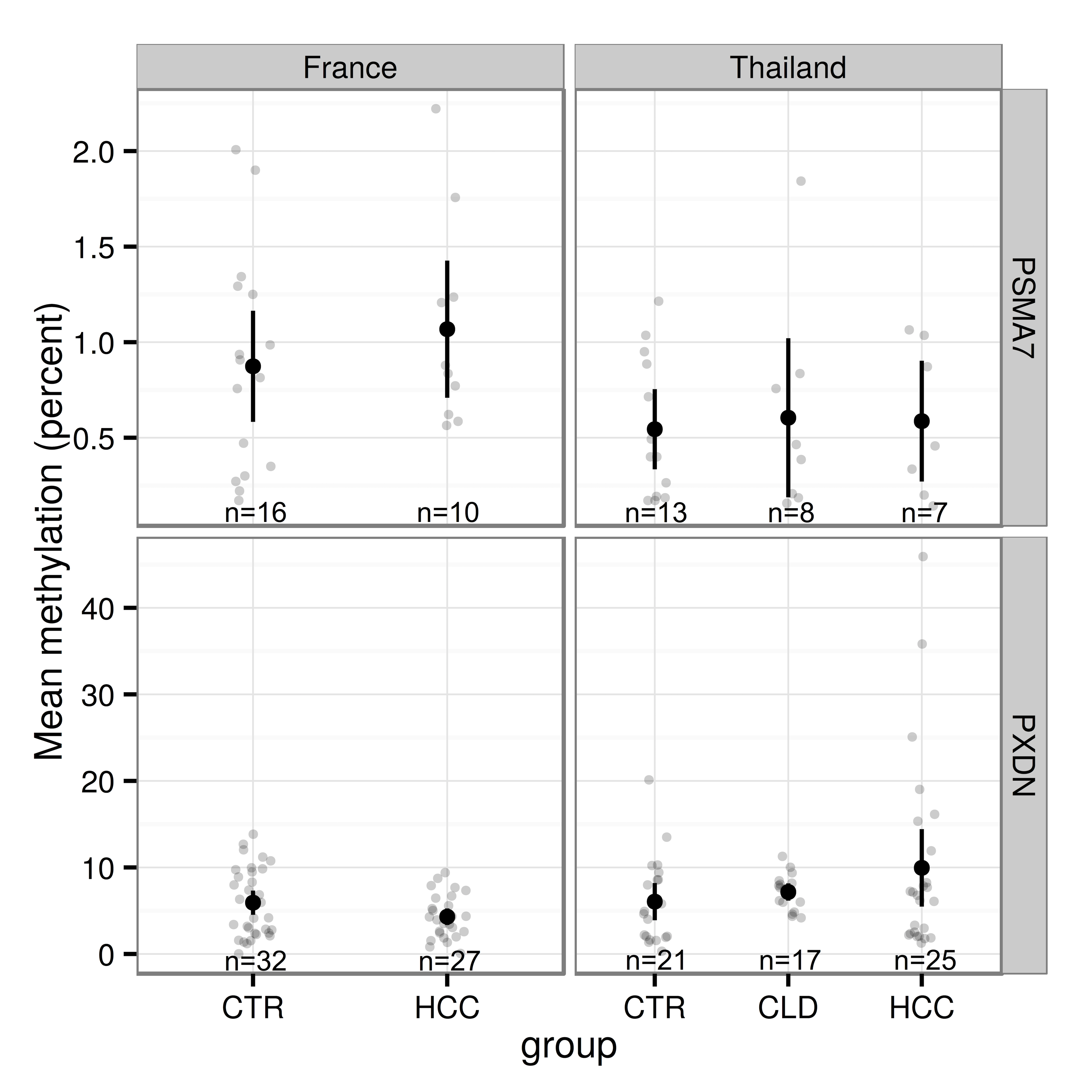

Supplement: S1 Fig — HCC = hepatocellular carcinoma, CTR = control, CLD = chronic liver disease. (TIFF) [file pone.0174265.s001.tiff]

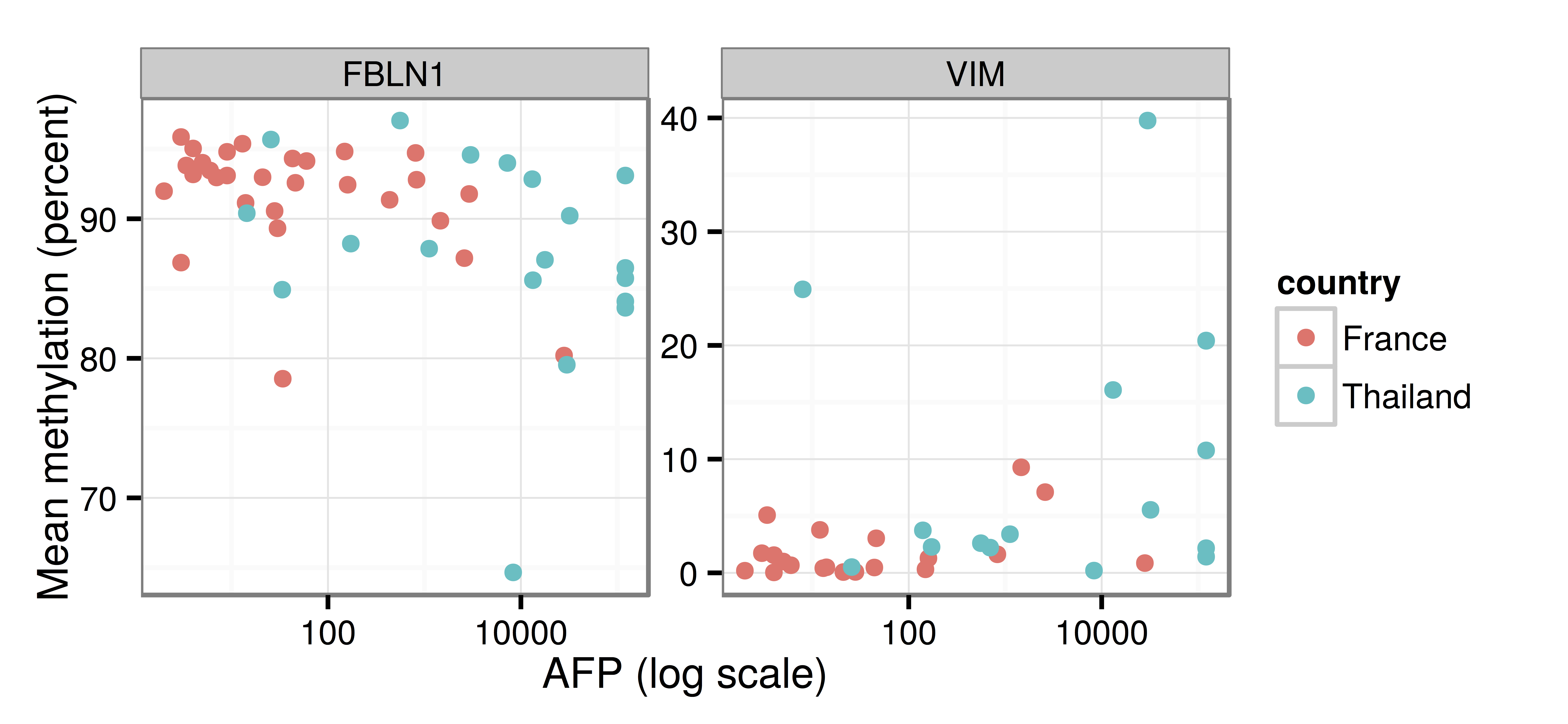

Supplement: S2 Fig — (TIFF) [file pone.0174265.s002.tiff]

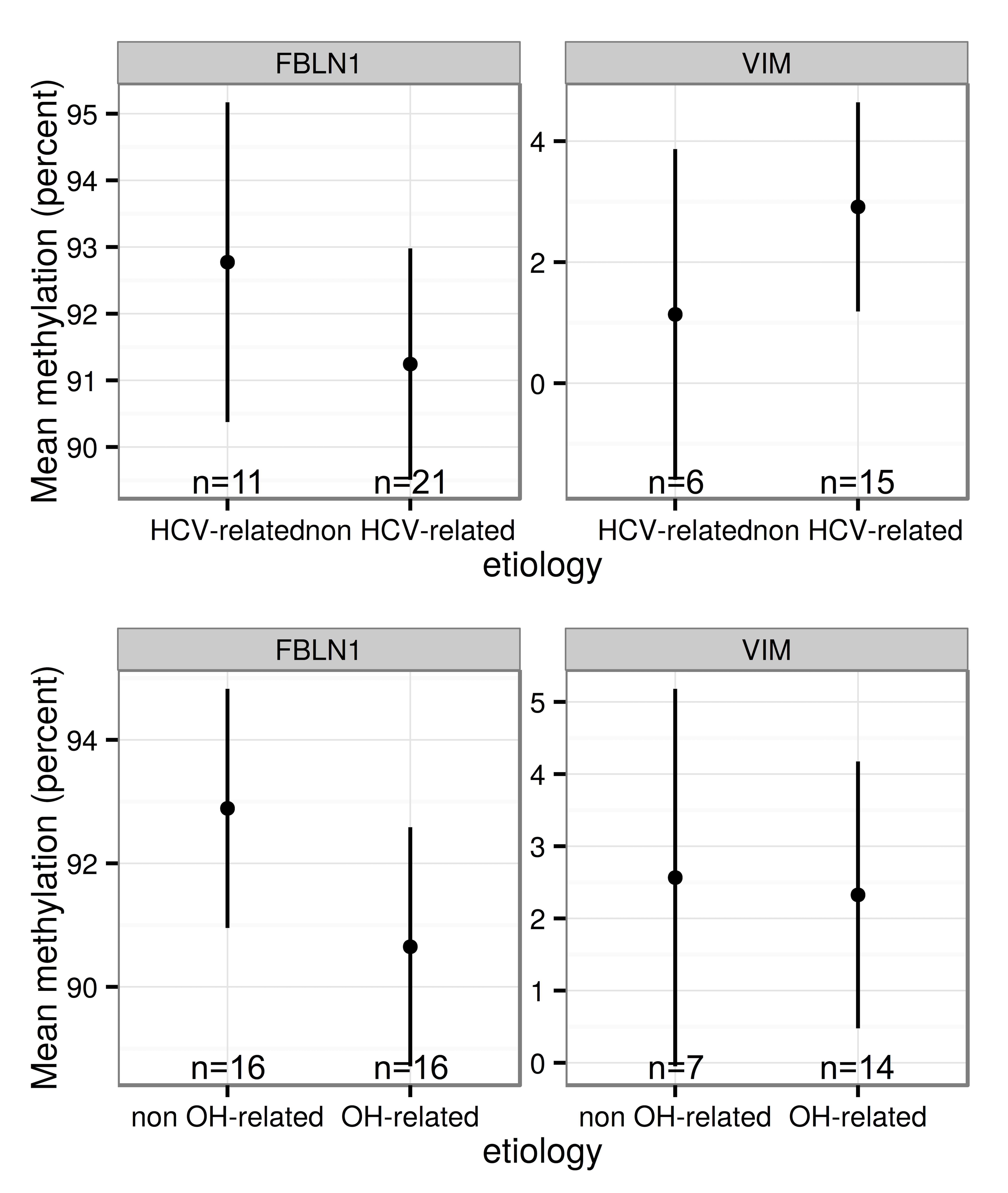

Supplement: S3 Fig — HCV = Hepatitis C virus, OH = alcohol. (TIFF) [file pone.0174265.s003.tiff]

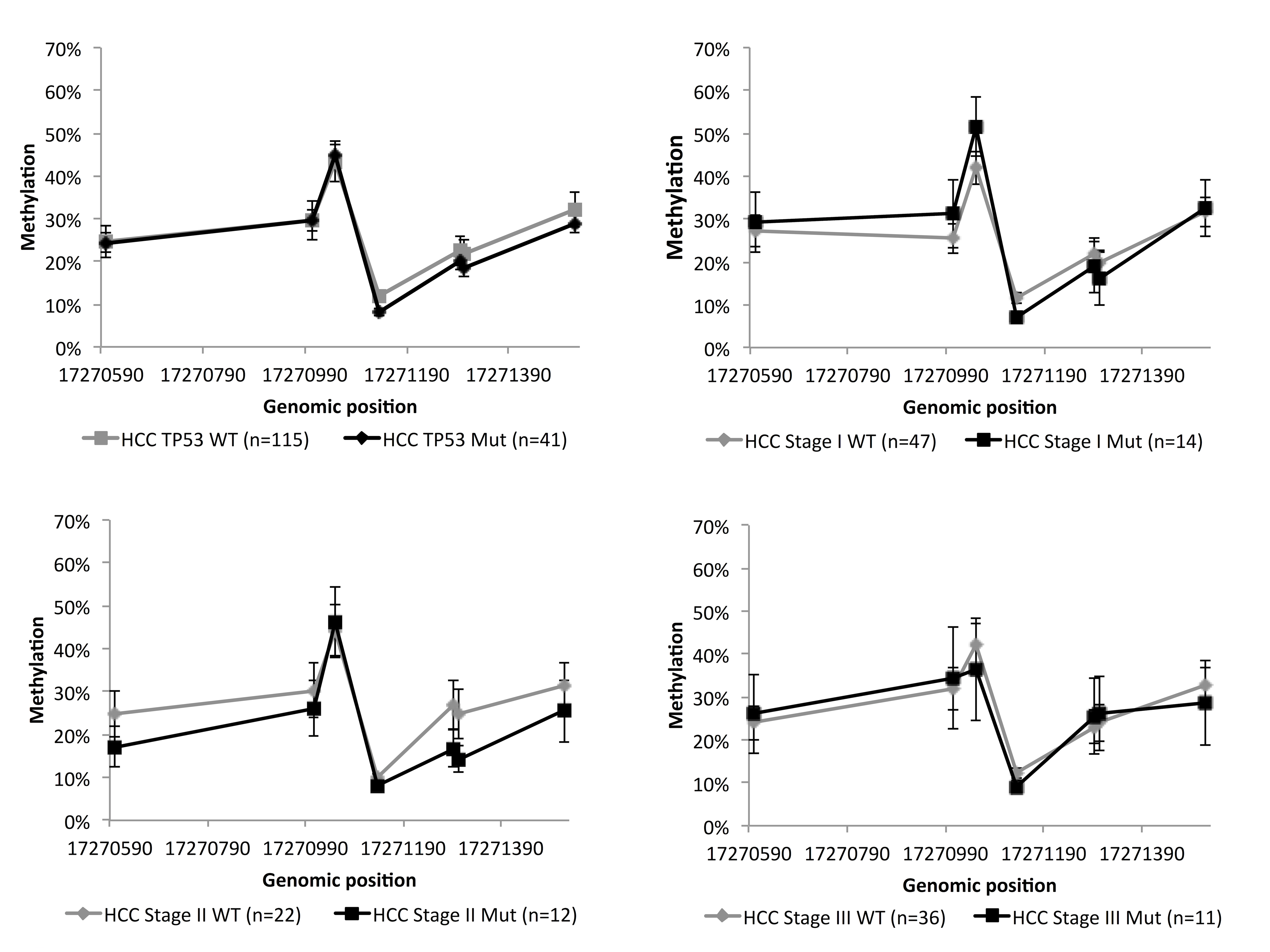

Supplement: S4 Fig — A) VIM methylation compared with overall TP53 mutation status, B) VIM methylation by TP53 mutation in stage I tumors, C) VIM methylation by TP53 mutation in stage II tumors, D) VIM methylation by TP53 mutation in stage III tumors. MUT = mutated, WT = wild type. (TIFF) [file pone.0174265.s004.tiff]
