# Supplementary material for: Targeted deep sequencing of plasma circulating cell-free DNA reveals Vimentin and Fibulin 1 as potential epigenetic biomarkers for hepatocellular carcinoma
Source: PLoS One. 2017 Mar 23;12(3):e0174265. doi: 10.1371/journal.pone.0174265 (PMC5363871; doi:10.1371/journal.pone.0174265)
Supplement: S1 Table — (DOCX) [file pone.0174265.s005.docx]

S1 Table. Characteristics of the French series

|  |  | Control | Hepatocellular carcinoma |
| --- | --- | --- | --- |
|  |  | n (%) | n (%) |
| Age | <40 | 25 (60) | 0 (0) |
|  | 40-49 | 10 (24) | 1 (2) |
|  | 50-59 | 6 (12) | 12 (29) |
|  | ≥60 | 1 (2) | 29 (69) |
|  |  |  |  |
| Sex | Men | 9 (21) | 36 (86) |
|  | Women | 33 (79) | 6 (14) |
|  |  |  |  |
| Hepatitis B (HBsAg) | Positive | 0 (0) | 1 (2) |
|  | Negative | 42 (100) | 41 (98) |
|  |  |  |  |
| Hepatitis C (HCVAb) | Positive | 0 (0) | 17 (36) |
|  | Negative | 42 (100) | 25 (64) |
|  |  |  |  |
| BCLC^a^ | A | 0 (0) | 19 (45) |
|  | B | 0 (0) | 7 (17) |
|  | C | 0 (0) | 14 (33) |
|  | NA | - | 2 (5) |
| Total |  | 42 | 42 |

^a^BCLC = Barcelona Clinic Liver Cancer staging
