# Supplementary material for: Targeted deep sequencing of plasma circulating cell-free DNA reveals Vimentin and Fibulin 1 as potential epigenetic biomarkers for hepatocellular carcinoma
Source: PLoS One. 2017 Mar 23;12(3):e0174265. doi: 10.1371/journal.pone.0174265 (PMC5363871; doi:10.1371/journal.pone.0174265)
Supplement: S2 Table — (DOCX) [file pone.0174265.s006.docx]

S2 Table. Population characteristics of the Thailand case-control study

|  |  | Controls | Chronic Liver Disease | Hepatocellular carcinoma |
| --- | --- | --- | --- | --- |
|  |  | n (%) | n (%) | n (%) |
| Age | <40 | 2 (5) | 0 (0) | 2 (5) |
|  | 40-49 | 16 (38) | 7 (27) | 6 (14) |
|  | 50-59 | 13 (31) | 13 (50) | 22 (52) |
|  | ≥60 | 11 (26) | 6 (23) | 12 (29) |
|  |  |  |  |  |
| Sex | Men | 27 (64) | 16 (62) | 32 (76) |
|  | Women | 15 (36) | 10 (38) | 10 (24) |
|  |  |  |  |  |
| Hepatitis B (HBsAg) | Positive | 0 (0) | 12 (46) | 12 (29) |
|  | Negative | 42 (100) | 14 (54) | 30 (71) |
|  |  |  |  |  |
|  |  |  |  |  |
| Hepatitis C (HCVAb) | Positive | 0 (0) | 3 (12) | 2 (5) |
|  | Negative | 42 (100) | 23 (88) | 40 (95) |
| Total |  | 42 | 26 | 42 |
