# Supplementary material for: Targeted deep sequencing of plasma circulating cell-free DNA reveals Vimentin and Fibulin 1 as potential epigenetic biomarkers for hepatocellular carcinoma
Source: PLoS One. 2017 Mar 23;12(3):e0174265. doi: 10.1371/journal.pone.0174265 (PMC5363871; doi:10.1371/journal.pone.0174265)
Supplement: S3 Table — (DOCX) [file pone.0174265.s007.docx]

S3 Table. Characteristics of the HCC tissue samples.

| Patient | Age | Gender | Etiology^a^ | EGS^b^ |
| --- | --- | --- | --- | --- |
|  |  |  |  |  |
| 1 | 70 | Male | NASH | 3 |
| 2 | 75 | Male | NASH | 2 |
| 3 | 68 | Female | NASH | 3 |
| 5 | 68 | Male | NASH | 2 |
| 6 | 70 | Male | Alcohol | 2 |
| 7 | 63 | Male | Alcohol | 2 |
| 8 | 74 | Male | Alcohol | 2 |
| 9 | 60 | Male | Alcohol | 3 |
| 10 | 63 | Male | Alcohol | 4 |

^a^NASH = Nonalcoholic fatty liver disease

^b^Edmondson-Steiner Grading system.
