# Supplementary material for: Targeted deep sequencing of plasma circulating cell-free DNA reveals Vimentin and Fibulin 1 as potential epigenetic biomarkers for hepatocellular carcinoma
Source: PLoS One. 2017 Mar 23;12(3):e0174265. doi: 10.1371/journal.pone.0174265 (PMC5363871; doi:10.1371/journal.pone.0174265)
Supplement: S4 Table — (DOCX) [file pone.0174265.s008.docx]

S4 Table. Primer and product sequences for the sequencing targets.

| **Gene** | **Primer F** | **Tm F** | **Primer R** | **Tm R** | **Product** | **Product (bp)** |
| --- | --- | --- | --- | --- | --- | --- |
| *FBLN1* | AGGTTTTTTAATTGGAGGAATTATGA | 50.6 | TAAATTCACCATAAACCCCTTAAAC | 50.8 | AGGTTTTTTAATTGGAGGAATTATGAATGTG***Y***GGTTATATGTTTTTTTGTTTTTTGGAGTATATGTTGTATG***Y***GTTTTTGTTGAGTGTAAGGTTGGG***Y***GGGG***Y***GT***Y***GGTGGTTTAAGGGGTTTATGGTGAATTTA | 135 |
| *HINT2* | AAATAGTTTTTTTTGTAGTAGGTGG | 49.2 | CCTCTTCAAATCTAATTCCTATTAATAC | 49.6 | AAATAGTTTTTTTTGTAGTAGGTGG***Y***GGAGTTT***Y***GTTTTTTTTTTTT***Y***G***Y***GTATTAATAGGAATTAGATTTGAAGAGG | 78 |
| *LAMC1* | GGTTATTAAGTTTTGTTATTTGTG | 46 | AATAAAACTAAAATACTACACCCC | 47.7 | GGTTATTAAGTTTTGTTATTTGTG***Y***GA***Y***GT***Y***GGGTAGTTTTATTTGTAGTA***Y***GGGGTAGTTTTTTTGAT***Y***GATTATAATAATTAGGT***Y***GATATTATTTGGTGGTAAAGTTAGATTATGTTGGT***Y***GGGGTGTAGTATTTTAGTTTTATT | 148 |
| *LTBP1* | GTAATTTTAGTGGATGTGGAGGGT | 53.3 | TATCAAACCTAAACAACCAATCTCC | 52.4 | GTAATTTTAGTGGATGTGGAGGGTGGYGG***Y***GYGTTTT***Y***GGGTTTTTYGTA***Y***GTTTTTTTTAATTAGGATTTGT***Y***GTTTGA***Y***GTTA***Y***GTTTGT***Y***GGAGATTGGTTGTTTAGGTTTGAT | 118 |
| *LTBP2* | TTGTAAGGTTGTAGTTTGGGTTTTT | 52.7 | CCTTTCTCCTACTCTAAACAAATTC | 50.6 | TTGTAAGGTTGTAGTTTGGGTTTTT***Y***GATTTAAAGA***Y***GGTTTTTGAGG***Y***GAGGGG***Y***GGGAGAA***Y***GATT***Y***GGGGTAGGTTTGTAGTT***Y***GAATTTGTTTAGAGTAGGAGAAAGG | 112 |
| *PSMA2* | GTTGATTATATTTAGGTATGGAAGGG | 50.6 | CCTACTAAACCCTCCAAAAACAC | 52.2 | GTTGATTATATTTAGGTATGGAAGGGGTTTT***Y***GAGGTTTTTAG***Y***GTAGT***Y***GAGGTTGTTGGTATTTTGT***Y***GATTTAGTTTTTTTTATTTTGA***Y***GT***Y***GGTATTGGTGTTTTTGGAGGGTTTAGTAGG | 126 |
| *PSMA7* | GGTTATTTTTTTTAAGTGGAGTA | 45.6 | ACCCTCTCTAAACTCCCCAAC | 53.8 | GGTTATTTTTTTTAAGTGGAGTA***Y***G***Y***GTAGGAGGT***Y***GTTAAGAAGGGTT***Y***GAT***Y***G***Y***GGTGAGG***Y***GG***Y***GGGGTTTG***Y***G***Y***GGGGT***Y***GGGGA***Y***G***Y***GGG***Y***GGAAGTGGGGTTGGGGAGTTTAGAGAGGGT | 126 |
| *PXDN* | GGTAGAGAGTAGGTGGTTGATAGGTT | 56.1 | CCCTCATTCCTTTTAAAAAAACTAAA | 50 | GGTAGAGAGTAGGTGGTTGATAGGTTT***Y***GGGGAGT***Y***GGAT***Y***GTTTGGGTTTAATTTTTT***Y***GTAGATTTTTTTGTTGTG***Y***GTTTTGGGGTTTGGGTTTTAGTTTTTTTAAAAGGAATGAGGG | 121 |
| *TGFB1* | TTTTTAGGGTTGAAGGGATTTTT | 49.7 | AAAAAAACATAAAAAAACTAAACCA | 46.4 | TTTTTAGGGTTGAAGGGATTTTTTT***Y***GGAGTT***Y***GTTTA***Y***G***Y***GAGATGAGGA***Y***GGTGGTTTAGTTTTTTTATGTTTTTTT | 79 |
| *UBE2L3* | TTTTAATTTTAGGAAGGTTTGAGGT | 50.2 | CCAAAAATAACACTTTATCAAAAAAAA | 48.2 | TTTTAATTTTAGGAAGGTTTGAGGT***Y***GGTTT***Y***GAT***Y***G***Y***GG***Y***G***Y***GGTTTTGTTTTTTTTTTTTTTATTTAGTTATTT***Y***GATTTTTTTTTTTTGATAAAGTGTTATTTTTGG | 110 |
| *VIM* | GGGATTATGTTTAGTTTTAGGTTT | 48 | AAAAATCCCCTCCCACTACC | 51.8 | GGGATTATGTTTAGTTTTAGGTTT***Y***GGAGTAGGAAGGTT***Y***GAGGG***Y***GTTTTTATTTTATT***Y***GTTTATTTTTTT***Y***GTTTTT***Y***GTTAGGTTTTTATTGGTTGG***Y***G***Y***GTTT***Y***G***Y***GGTTGGGATGGTAGTGGGAGGGGATTTTT | 140 |
| *YWHAZ* | TATTTGGGTAGGAAAATTTAGTTTA | 47.5 | ACTAATCCTAACAACCTTAACCC | 50.7 | TATTTGGGTAGGAAAATTTAGTTTA***Y***GAGAAA***Y***GGGTT***Y***GTG***Y***GGTTA***Y***GTTTGATTGTTTGTTTT***Y***GGGTTAAGGTTGTTAGGATTAGT | 90 |
