# Supplementary material for: Targeted deep sequencing of plasma circulating cell-free DNA reveals Vimentin and Fibulin 1 as potential epigenetic biomarkers for hepatocellular carcinoma
Source: PLoS One. 2017 Mar 23;12(3):e0174265. doi: 10.1371/journal.pone.0174265 (PMC5363871; doi:10.1371/journal.pone.0174265)
Supplement: S1 File — (DOCX) [file pone.0174265.s009.docx]

**Cell lines and cell culture**

HepG2, HepG2.2.15, Hep3B and PLC/PRF/5 cells were obtained from the American Type Culture Collection (USA) and Mahlavu cell development was described previously (1). Cells were cultured at 37C under 5% CO2, in minimum essential medium (Gibco-Invitrogen) supplemented with 10% fetal calf serum (Gibco-Invitrogen), 100 UI/ml penicillin/100 lg/ml streptomycin /2 mM glutamin (Sigma–Aldrich), non-essential amino acids and 1mM sodium pyruvate (both from Invitrogen). The development of HepaRG cells has been previously described (2). Cells were grown for two weeks at 37 °C, 5% CO2 in proliferation medium (Williams’E medium (Gibco-Invitrogen), 10% non-decomplemented Fetal Calf Serum (Hyclone Fetalclone II, Thermo Scientific), 2 mM glutamine (Gibco-Invitrogen), 50 U ml^-1^ penicillin (Gibco-Invitrogen), 50 U ml^-1^ streptomycin (Gibco-Invitrogen), 5 μg ml^-1^ human recombinant insulin (Sigma-Aldrich), 0.5 μM dexamethasone (Sigma-Aldrich), 20 μg ml^-1^ gentamycine (Gibco-Invitrogen). For differentiation, cells were grown for two additional weeks at 37 °C, 5% CO 2 in proliferation medium supplemented with 1.8% DMSO (Sigma-Aldrich) and 5 ng ml^-1^ Epidermal Growth Factor (AbCys). At the end of the experimental period, cells were washed in PBS and pellets were frozen at -80°C.

References

1. Oefinger PE, Bronson DL, Dreesman GR. Induction of hepatitis B surface antigen in human hepatoma-derived cell lines. The Journal of general virology. 1981;53(Pt 1):105-13.

2. Gripon P, Rumin S, Urban S, Le Seyec J, Glaise D, Cannie I, et al. Infection of a human hepatoma cell line by hepatitis B virus. Proc Natl Acad Sci U S A. 2002;99(24):15655-60.
